# Meta-Analysis of Effectiveness and Safety of Botulinum Toxin in the Treatment of Complex Regional Pain Syndrome

**DOI:** 10.3390/life12122037

**Published:** 2022-12-06

**Authors:** Yu-Chi Su, Pei-Chun Hsieh, Yao-Hong Guo, Yu-Ching Lin

**Affiliations:** 1 Department of Physical Medicine and Rehabilitation, National Cheng Kung University Hospital, College of Medicine, National Cheng Kung University, Tainan 70101, Taiwan; 2 Department of Physical Medicine and Rehabilitation, College of Medicine, National Cheng Kung University, Tainan 70101, Taiwan

**Keywords:** complex regional pain syndrome, botulinum toxin, meta-analysis, lumbar sympathetic block

## Abstract

Complex regional pain syndrome (CRPS) is characterized by pain, limited range of motion, swelling, skin changes, vasomotor instability, and patchy bone demineralization. Conservative management strategies for CRPS include physical and occupational therapy, psychosocial and behavioral therapy, and pharmacotherapy. However, some patients still experience CRPS symptoms after receiving conventional treatments. Therefore, botulinum toxin (BoNT) has been applied to patients with CRPS in several trials considering its analgesic effect in musculoskeletal and neuropathic pain; however, the results were controversial. We conducted the study to explore the effectiveness and safety of BoNT in patients with complex regional pain syndrome (CRPS). A search was performed using the following electronic databases up to 19 October 2022: PubMed, Embase, and Cochrane Library. We included both randomized controlled trials and nonrandomized controlled studies involving patients with complex regional pain syndrome managed with botulinum toxin. Cochrane risk-of-bias tool and Joanna Briggs Institute Critical Appraisal Checklist were used for quality assessment for randomized controlled trials and quasi-experimental studies. Only randomized controlled trials entered the meta-analysis. The primary outcome was the visual analogue scale of pain presented as a weighted mean difference (WMD) and 95% confidence interval (CI). The secondary outcome was the risk of adverse events presented as an odds ratio (OR) with 95% CI. We analyzed eight articles with 176 patients, including three randomized controlled trials with 62 participants. The age of the patients ranged from 23.8 to 51 years old. The duration of the disease ranged from 2.2 to 11.8 years. The proportion of females ranged from 16.6% to 100%. The route of administration of BoNT included: (1) lumbar sympathetic block (LSB), (2) intramuscular injection, (3) subcutaneous or intradermal injection (SC/ID). Improvement in pain was revealed in six studies, and adverse events were all self-limited and temporary. Meta-analysis revealed a significant reduction in pain at the first follow-up between 3 weeks to 1 month after intervention (WMD, −1.036, 95% CI, −1.673 to −0.400) but not at the second follow-up between 2 to 3 months after treatment (WMD, −0.895, 95% CI, −2.249 to 0.458). Subgroup analyses between LSB and SC/ID were nonsignificant at both follow-up periods (*p* = 0.422, 0.139). The risk of adverse events was similar between the BoNT and control group (OR, 0.698, 95% CI, 0.136 to 3.581). In conclusion, BoNT may be effective and safe for alleviating pain in patients with CRPS. However, we could not draw definite conclusions due to small sample size and high between-study heterogeneity. The limited number of participants may conceal the possibility of serious adverse events. Further large-scale randomized controlled trials are warranted to delineate the role of BoNT in CRPS.

## 1. Introduction

Complex regional pain syndrome (CRPS) is characterized by pain, limited range of motion, swelling, skin changes, vasomotor instability, and patchy bone demineralization [1,2]. Moreover, approximately 20% of patients with CRPS develop fixed dystonia [3,4]. There are three subtypes of CRPS [5]. In type 1 CRPS, no nerve injury can be identified, while type 2 CRPS arises after an injury or trauma to a peripheral nerve. A third class, named CRPS not otherwise specified, refers to those who partially meet CRPS criteria and are not better explained by any other condition [5]. So far, the pathogenesis of CRPS is still unclear [6]. Several diagnostic criteria have been proposed, including the earlier Orlando criteria and the current Budapest criteria [7].

Conservative management strategies for CRPS include physical and occupational therapy, psychosocial and behavioral therapy, and pharmacotherapy [7,8]. A review published in 2020 concluded that physical therapy with occupational and cognitive behavioral treatment is the recommended option for pediatric CPRS, while pharmacological therapy should be reserved for refractory conditions and selected individuals [9]. Further interventional procedures encompass trigger point injections, regional sympathetic nerve blocks, spinal cord stimulation, and sympathectomy [10]. However, some patients still experience CRPS symptoms after receiving conventional treatments [11,12]; hence, identifying new methods to treat this group of patients is necessary.

In previous research [13,14,15], botulinum toxin (BoNT) has been demonstrated to have an analgesic effect in musculoskeletal and neuropathic pain. The mechanisms might be inhibition of the release of proinflammatory and pain mediators [15]. The pathophysiology of CRPS remains undetermined, but several studies have demonstrated a substantial increase in proinflammatory cytokines in affected tissue, plasma, and cerebrospinal fluid in individuals with CRPS [16,17,18,19]. Theoretically, BoNT may alleviate pain in CRPS through the control of proinflammatory factors [20] and pain [21]. In addition, it may treat dystonia by blocking the cholinergic innervation of striate muscles [22]; hence, BoNT possesses the potential to relieve pain and dystonic symptoms related to CRPS. Several routes of administration with BoNT, such as intra-muscular, subcutaneous and lumbar sympathetic block (LSB), have been surveyed in previous articles [23,24,25,26,27,28,29], and the results of the effectiveness of BoNT were controversial. However, no research has addressed the topic of BoNT in CRPS comprehensively so far.

The objective of our study was to assess the effectiveness and safety of BoNT in CRPS by performing a meta-analysis of articles on the PubMed, Embase, and Cochrane Library databases between their inception and 19 October 2022. Both randomized controlled trials and quasi-experimental studies were included. We also compared the effectiveness of BoNT between different delivery routes.

## 2. Materials and Methods

This meta-analysis was performed according to the Preferred Reporting Items for Systematic Review and Meta-Analysis (PRISMA) guidelines (see File S1 for the PRISMA checklist) [30]. The protocol was registered on the International Platform of Registered Systematic Review and Meta-analysis Protocols (INPLASY2022100087).

### 2.1. Eligibility Criteria

The inclusion criteria were as follows: (1) case arm consisting of patients with CRPS treated with BoNT, (2) without control arm or control arm with treatment different from the case arm, and (3) randomized controlled trials or quasi-experimental studies. The exclusion criteria were as follows: (1) studies that did not report the administration route of BoNT, (2) case reports and conference proceedings. No restrictions were set for publication language and the center where the studies were conducted.

### 2.2. Search Strategy

We retrieved papers from the PubMed, Embase, and Cochrane Central Register of Controlled Trials. We searched the online literature using the following combination of subject heading search terms: “complex regional pain syndrome” and “botulinum toxin.” The search period was from the inception of the database to 19 October 2022 (see File S1 for the full search strategy).

### 2.3. Study Selection and Data Extraction

After excluding duplicate articles, the first two authors independently reviewed the titles and abstracts of applicable studies. If consensus was not reached after discussion, the opinion of the senior author (YCL) was sought. We used a data collection form to record the extracted data from the included studies on the following: first author, year of publication, participants’ demographics and clinical presentations, BoNT dosage, BoNT dilution method, commercial form of BoNT, injection technique, comparative regimen, clinical assessments and electrophysiological outcomes. For data presented as charts, we used ImageJ software (ImageJ v1.52k by Wayne Rasband, NIH, Bethesda, MD, USA) “http://wsr.imagej.net/distros/win/ij152-win-java8.zip (accessed on 15 January 2019)” to extract the information [31]. The authors were approached to resolve uncertainties.

### 2.4. Quality Assessment

The risk of bias was assessed using the Cochrane risk-of-bias tool [32] for randomized controlled trials. The quality of the nonrandomized controlled studies was evaluated using the Joanna Briggs Institute Critical Appraisal Checklist for Quasi-Experimental Studies [33]. Disagreements about the results were resolved through discussion, and the final results were determined by the senior author if consensus could not be reached. The results are presented using Reviewer Manager version 5.3 (Cochrane, London, UK).

### 2.5. Statistical Analysis

To increase the robustness of the results of the meta-analysis, only randomized controlled trials were included in the meta-analysis. The primary outcome was the intensity of pain in patients with CRPS after treatment with BoNT. The visual analog scale pain scores for the intervention and control groups were first extracted. Meta-analysis was then conducted for the pain scores at different follow-up time points. The results were represented as weighted mean differences (WMD) and 95% confidence intervals (CI). The secondary outcome was the risk of adverse events after intervention. The number of adverse events in the control and BoNT group were extracted, and the results were represented by odds ratio (OR) and 95% CIs. The effect size was pooled using a random effects model. The I^2^ value was used to grade between-study heterogeneity, and statistical significance was reached when I^2^ exceeded 50% [34]. Subgroup analysis was conducted for different administration routes of BoNT, and significant difference between subgroups was identified when *p* < 0.05. Funnel plots and the Egger test were adopted for publication bias [35]. Statistical significance was defined as a two-tailed *p* value of <0.1 [36]. Sensitivity analysis was also performed by excluding the studies that did not score low risk of bias in all of the categories during quality assessment. We used Comprehensive Meta-analysis Software version 3 (Biostat, Englewood, NJ, USA) for the analysis.

### 2.6. Certainty of Evidence

The certainty of the evidence of outcomes were assessed by the Grading of Recommendations Assessment, Development and Evaluation (GRADE) methodology. Our meta-analysis included randomized controlled trials only, so the results start as high certainty. The final rating depends on the publication bias, indirectness, overall risk of bias, imprecision, and inconsistency [37].

## 3. Results

### 3.1. Study Selection and Description

**Figure 1:**
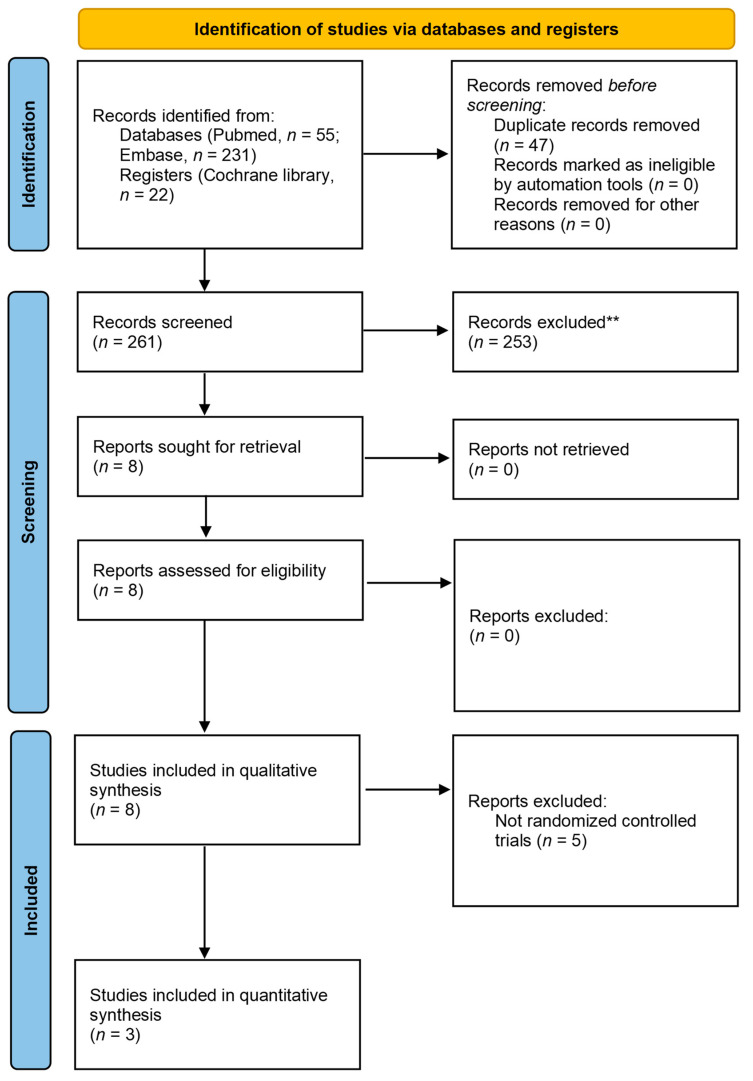
Literature screening process and results.

In the initial search, 308 articles were obtained, of which 8 fulfilled our inclusion criteria. The details are presented in the PRISMA flowchart (Figure 1). Four of them were uncontrolled longitudinal studies [23,25,26,28], three were randomized controlled trials [24,27,38], and one was a retrospective cohort study [29]. All of the papers comprised at least one arm using BoNT type A. The main characteristics of the papers included are listed in Table 1.

Three articles applied BoNT intramuscularly in patients with CRPS-related dystonia [25,26,28]. Of these, Cordivari et al. [28] revealed that both pain and dystonia improved after treatment, Kharkar et al. [25] reported an analgesic effect, and Schilder et al. [26] concluded that BoNT relieved dystonia, which they measured using surface electromyography.

Two studies administered BoNT intradermally or subcutaneously [23,24]. Safarpour et al. [24] concluded that BoNT failed to alleviate pain, and the procedure was intolerable because of the extreme level of discomfort. Lessard et al. [23] performed nerve blocks before BoNT treatments to increase injection tolerance and revealed a significant decrease in pain after multiple sessions of monthly BoNT treatment, with maximum pain reduction being achieved by the ninth treatment session.

Three articles performed lumbar sympathetic block with a combination of local anesthetic and BoNT [27,29,38]. Carroll et al. [27] concluded that additional BoNT prolonged the analgesic effect of standard LSB, and Lee et al. [29] revealed that adding BoNT type B to the local anesthetic prolonged the effect of LSB more than adding BoNT type A. Yoo et al. [38] found that LSB with BoNT type A increased the temperature and reduced the pain of the affected foot. Further details of the intervention protocols are listed in Table 2.

Of the 8 studies included, 4 reported transient side effects. Carroll et al. [27] noted one individual with transient nausea and emesis after LSB with a combination of BoNT and local anesthetic. Lee et al. [29] reported that one patient had transient dizziness after LSB with local anesthetic and BoNT type A and one patient had temporary dizziness after LSB with local anesthetic and BoNT type B. Kharkar et al. [25] revealed that one patient had a transient neck drop after an intramuscular injection of BoNT type A. Yoo et al. [38] found six participants in the control group and three in the BoNT group had mild post-procedure dizziness. No serious adverse events were reported in the included articles.

### 3.2. Risk-of-Bias Assessment

**Figure 2:**
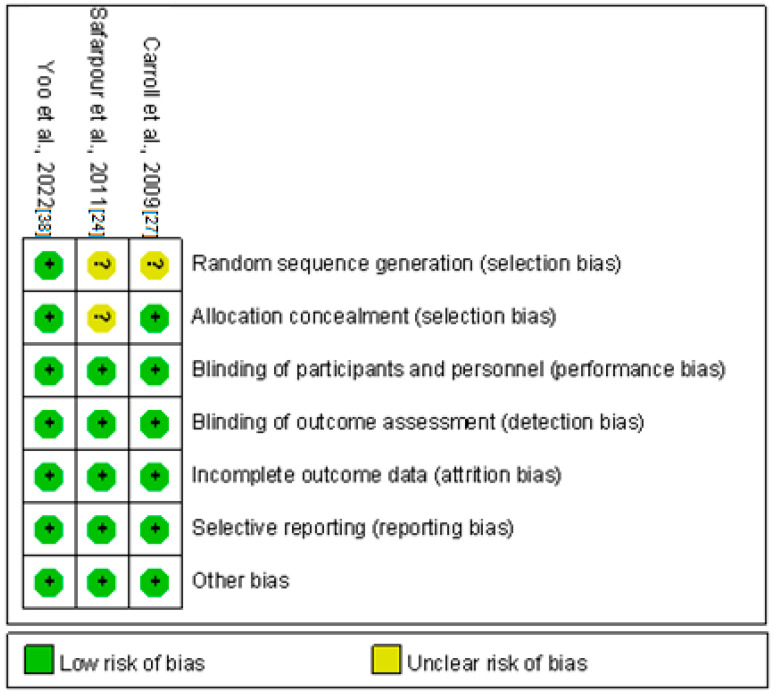
Summary graph for risk of bias of randomized controlled trials.

**Figure 3:**
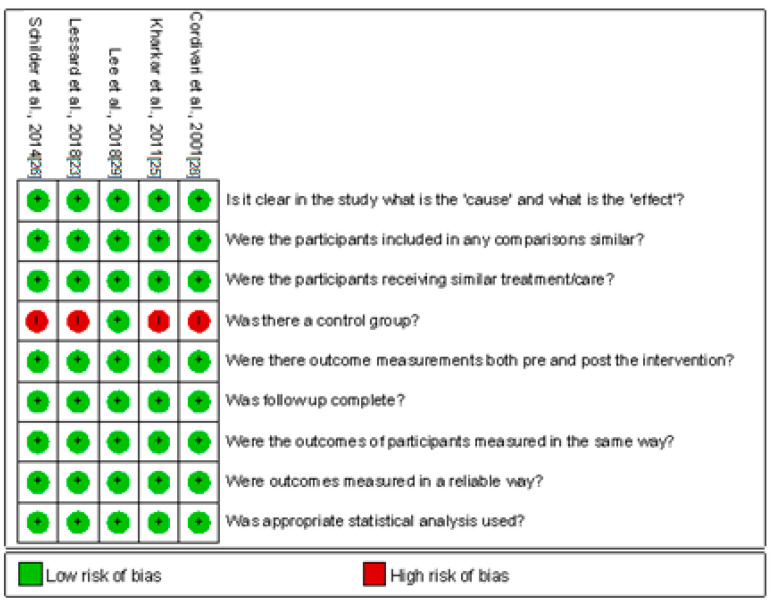
Summary graph for risk of bias of quasi-experimental studies.

Two [24,27] of the three randomized controlled trials [24,27,38] had an unclear risk of selection bias because neither provided details on the generation of random sequences. Furthermore, Safarpour et al. [24] did not detail the allocation concealment (Figure 2). Of the 5 nonrandomized controlled studies, Schilder et al. [26], Cordivari et al. [28], Kharkar et al. [25], and Lessard et al. [23] did not include a control group (Figure 3).

### 3.3. Outcomes

**Figure 4:**
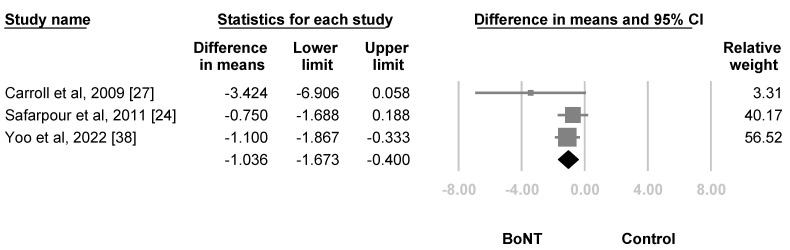
Forest plot of the weighted mean difference in the reduction of VAS scores 3 weeks to 1 months after treatment. Squares were individual studies, lines were 95% confidence intervals, and the diamond was the summary effect size.

**Figure 5:**
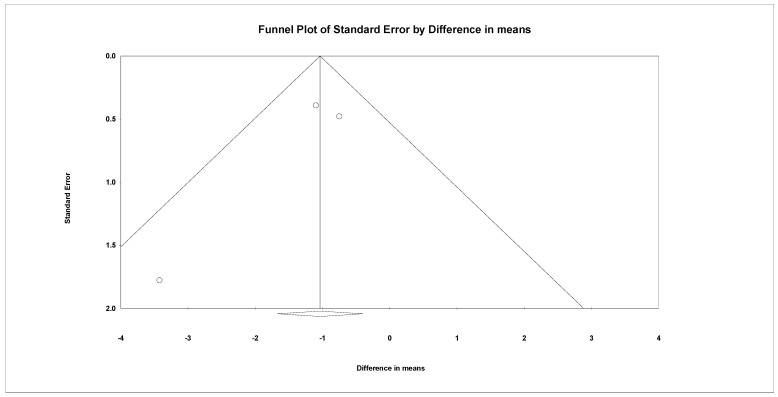
Funnel plot of the weighted mean difference in the reduction of VAS scores 3 weeks to 1 months after treatment. Dots indicate individual studies, and the diamond indicates the summary effect size.

The first follow-up for pain ranged from 3 weeks to 1 month after BoNT. The meta-analysis included two articles using LSB [27,38] and one using subcutaneous and intradermal injection [24]. The summarized effect size revealed a significantly larger decrease of VAS score in the BoNT group than the control group (WMD, −1.036, 95% CI, −1.673 to −0.400, I^2^ = 8.713%, Figure 4). For subgroup analysis, the difference between LSB (WMD, −1.616, 95% CI, −3.509 to 0.277, I^2^ = 38.748%) and subcutaneous and intradermal injection (WMD, −0.750, 95% CI, −1.688 to 0.188, I^2^ = 0%) did not reach statistical significance (*p* = 0.422). For sensitivity analysis, after excluding the studies which did not score a low risk of bias in all of the categories during quality assessment, the estimation point was still significant (WMD, −1.100, 95% CI, −1.867 to −0.333, I^2^ = 0%). No significant publication bias was detected by the funnel plot and Egger’s test (*p* = 0.36854, Figure 5).

**Figure 6:**
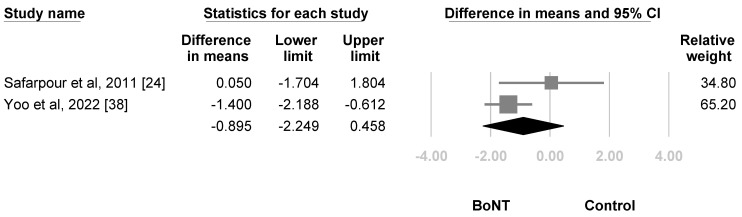
Forest plot of the weighted mean difference in the reduction of VAS scores 2 months to 3 months after treatment. Squares were individual studies, lines were 95% confidence intervals, and the diamond was the summary effect size.

The second follow-up for pain ranged from 1 months to 2 months. The estimation point was nonsignificant (WMD, −0.895, 95% CI, −2.249 to 0.458, I^2^ = 54.214%, Figure 6). For subgroup analysis, the difference between LSB (WMD, −1.400, 95% CI, −2.188 to −0.612, I^2^ = 0%) and subcutaneous and intradermal injection (WMD, 0.050, 95% CI, −1.704 to 1.804, I^2^ = 0%) did not reach statistical significance. (*p* = 0.139). For sensitivity analysis, the effect size turned significant (WMD, −1.400, 95% CI, −2.188 to −0.612, I^2^ = 0%) after excluding the studies that did not score low risk of bias in all of the categories during quality assessment. The funnel plot and Egger’s test were not conducted due to insufficient number of articles.

**Figure 7:**
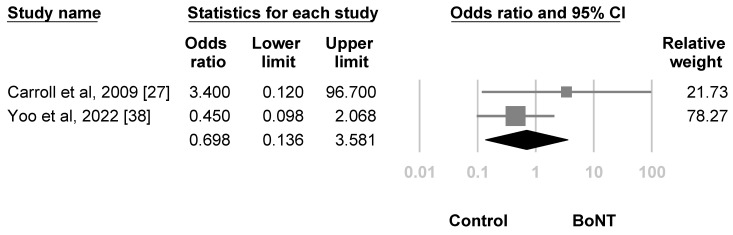
Forest plot of the risk of adverse events after treatment. Squares were individual studies, lines were 95% confidence intervals, and the diamond was the summary effect size.

For the risk of adverse events, the meta-analysis included two randomized controlled trials [27,38] using LSB. The difference between the BoNT group and the control group did not reach statistical significance (OR, 0.698, 95% CI, 0.136 to 3.581, I^2^ = 13.850%, Figure 7). For sensitivity analysis, the estimation point was still nonsignificant (OR, 0.450, 95% CI, 0.098 to 2.068, I^2^ = 0%) after excluding the studies that did not score low risk of bias in all of the categories during quality assessment. The subgroup analysis, funnel plot and Egger’s test were not conducted due to insufficient number of articles.

### 3.4. Certainty of Evidence

The certainty of the evidence of pain and risk for adverse events scored low to very low quality of evidence. The level was mainly downgraded because of the risk of bias and imprecision due to large 95% CI (Table 3).

## 4. Discussion

Our study found that the current evidence of BoNT in CRPS mainly focused on the augmentation of lumbar sympathetic block with BoNT. The meta-analysis revealed that BoNT decreased pain after 3 weeks to 1 months after BoNT injection, though the difference were not significant during the second follow-up at 2 to 3 months. The subgroup analysis showed that different administration routes did not alter the analgesic effect of BoNT. No serious adverse events were reported so far, and the meta-analysis revealed that adding BoNT to LSB did not increase the risk of adverse events.

The summary effect of our meta-analysis agreed with our initial hypothesis. The effect on pain at 3 weeks to 1 month not only reached statistical significance but also had clinical significance [39]. Additionally, the low between-study heterogeneity may imply a common analgesic mechanism despite the different administration routes. A previous research proposed that BoNT might possess a central antinociceptive effect through retrograde axonal transport [40]. BoNT injected subcutaneously may undergo such a pathway; thus, it can achieve similar results to LSB with BoNT. Moreover, our meta-analysis only included randomized controlled trials; the results were therefore robust at the evidence level of this meta-analysis. However, because of the small number of included studies, the possibility of false negatives cannot be excluded. The findings should be interpreted with caution.

Of the eight articles enrolled in our study, only one by Safarpour et al. concluded that BoNT was unable to relieve pain in patients with CRPS [24]. This might be the result of the low statistical power, dose of BoNT per session, or total number of treatment sessions. The trial of Safarpour et al. had a small sample size, with just eight patients recruited for their randomized controlled trial, making the findings susceptible to type II error [24]. With regard to BoNT dosage, Lessard et al. used a dose two times that of the dose of Safarpour et al. Moreover, Lessard et al. had multiple treatment sessions, but Safarpour et al. measured their outcome after a single session [23,24]. These differences may indicate that a higher dose per session and multiple sessions may increase the effectiveness of the treatment. However, we were unable to demonstrate our hypothesis quantitatively because of the insufficient number of trials. Future studies are necessary to verify the optimal dose and number of treatment sessions.

Cordivari et al. [28] and Kharkar et al. [25] reported that CRPS pain was alleviated after intramuscular interventions with BoNT type A, but these articles only included patients with CRPS and dystonia. No study addressed intramuscular injection in patients with CRPS without dystonia. However, some evidence has been reported for the effectiveness of the intramuscular administration of BoNT in alleviating pain that is not related to dystonia, such as trigeminal neuralgia [41], lateral epicondylitis [42], and migraine [43]. Because dystonia occurs only in approximately 20% of patients with CRPS [3,4], establishing whether BoNT is effective in CRPS without dystonia is warranted.

Despite the statistically and clinically significant results shown in our meta-analysis, the application of BoNT in CRPS still needs further research. Considering that CRPS is currently a diagnosis without objective data but with criteria involving ambiguous signs and symptoms, the indiscriminate use of pain medications to treat subjective symptoms of CRPS can be a risk factor for analgesic misuse or abuse [44]. The same situation is also applicable to children with CRPS. The diverse pathophysiology provides a variety of therapeutic targets, but there is still insufficient evidence to identify reliably effective pharmacotherapy for these patients [45].

Our meta-analysis has several strengths. First, this is the first article to systematically review the role of BoNT in CRPS. Second, we were the first to conduct a meta-analysis not only to summarize the effect size statistically but also to investigate the effectiveness of different administration routes. Third, we were the first to summarize the adverse events of BoNT in CRPS.

There are several limitations in our review. First, three studies [23,24,28] did not report the type of CRPS of their participants, making it difficult to know whether a different treatment efficacy existed between type I and type II of CRPS. Second, only one article [19] provided details of the symptoms and indications used to diagnose CRPS; thus, the relationship between treatment effectiveness and disease presentation was difficult to assess. Third, three papers [23,25,29] did not report the mean disease duration before BoNT treatment. This information is important because acute and chronic CRPS respond differently to conventional treatments [11]. Fourth, only two studies [25,27] have verified that no treatment modifications occurred during the trial, making it difficult to exclude the possibility of interference resulting from adjustments to the baseline medication. Fifth, two articles did not mention the commercial forms of BoNT adopted in their trials [25,27]. Studies have previously revealed that each BoNT formula has different properties, which are not interchangeable because of the disparity in manufacturing processes and bioassay methods for defining potency [46,47,48]. Future trials should clarify the commercial form of BoNT used in the trials to increase the reproducibility of the results. Sixth, six trials [23,24,25,27,28,29] included pain as an outcome measure, but only two [27,29] followed up on pain until it returned to baseline. The other experiments reported improvements in pain after intervention, but the follow-up time was relatively short, ranging from 4 weeks to 3 months. The long-term efficacy of such treatments requires further investigation. Seventh, no standard protocol for administering a BoNT injection to patients with CRPS currently exists; three different methods including intramuscular, subcutaneous or intradermal, and combined with a lumbar block, were identified in the literature. The high heterogeneity made it difficult to compare the treatment effects between studies, which may decrease the generalizability of our meta-analysis results. Eighth, four of the seven papers included in this review were case series. The lack of a control group makes it difficult to preclude a placebo effect [49]. Finally, the adverse events reported so far were temporary. However, the number of participants was relatively low, and recognizing serious adverse events may therefore be difficult [50].

## 5. Conclusions

In conclusion, BoNT may be effective and safe for alleviating pain in patients with CRPS. However, we could not draw definite conclusions due to small sample size and high between-study heterogeneity, such as treatment protocols, optimal injection dosage and timing of BoNT interventions. The limited number of participants may conceal the possibility of serious adverse events. Further large-scale randomized controlled trials are warranted to delineate the role of BoNT in CRPS.

## Figures and Tables

**Table 1:** Characteristics of enrolled articles.

Research	Country	Study Design	Age (Mean, Years)	Number of Cases of CRPS Type 1/2	Mean of Disease Duration (Years)	Gender (Male/Female)	Last Follow-Up	Other Treatments Besides Intervention
Cordivari et al., 2001 [28]	United Kingdom	Uncontrolled longitudinal study (case series)	42	NR	5.2	0/4	NR	NR
Carroll et al., 2009 [27]	United states	Crossover, randomized controlled trial	51	7/0	2.7	1/6	300 days	Continue medication started before the trial, no new therapy started in trial
Kharkar et al., 2011 [25]	United states	Uncontrolled longitudinal study (case series)	NR	26/11	NR	2/35	4 weeks	No significant change
Safarpour et al., 2011 [24]	United states	Randomized controlled trial	47	NR	5.5	3/5	2 months	NR
Schilder et al., 2014 [26]	Netherlands	Uncontrolled longitudinal study (case series)	42	17/0	11.8	2/15	5 weeks	NR
Lee et al., 2018 [29]	Korea	Retrospective cohort	BoNT-A: 26 BoNT-B: 23	18/0	NR	BoNT-A: 5/0 BoNT-B: 10/3	56 days	All patients show over 50% reduction in pain after LSB with ropivacaine before.
Lessard et al., 2018 [23]	Canada	Uncontrolled longitudinal study (case series)	50	NR	NR	12/8	12 months	Pain refractory to 2 or more treatments
Yoo et al., 2022 [38]	Korea	Randomized controlled trial	Intervention: 44.8 Control: 43.7	Intervention: 20/3 Control: 22/2	Intervention: 2.225 Control: 2.1	Intervention: 12/11 Control: 12/12	3 months	Pain duration of at least 6 months despite conventional pain management; confirmed temperature increase during the screening lumbar sympathetic ganglion block

BoNT-A: botulinum neurotoxin type A; BoNT-B: botulinum neurotoxin type B; NR: not reported.

**Table 2:** Extracted data from enrolled articles.

Research	Treatment Sessions	Interval of Intervention	Commercial Forms	Injection Dose (Unit)	Injection Site	Tool for Injection Guidance	Outcomes
Cordivari et al., 2001 [28]	7.4 (mean)	3–6 (month)	abobotulinumtoxinA	450–1200	Intramuscular	Electromyography	Pain, dystonia, function, posture
Carroll et al., 2009 [27]	1	NR	Intervention: BoNT-A with bupivacaine; Control: bupivacaine	75	LSB	Fluoroscopy	Pain, adverse events
Kharkar et al., 2011 [25]	1	NR	BoNT-A	100	Intramuscular	Electromyography	Pain, adverse events
Safarpour et al., 2011 [24]	1	NR	Intervention: onabotulinumtoxinA; Control: normal saline	40–200	Intradermal/subcutaneous	NR	Pain
Schilder et al., 2014 [26]	1	NR	onabotulinumtoxinA	20	Intramuscular	NR	CMAP
Lee et al., 2018 [29]	1	NR	onabotulinumtoxinA; rimabotulinumtoxinB	BoNT-A: 100; BoNT-B: 5000	LSB	Fluoroscopy	Pain, adverse events
Lessard et al., 2018 [23]	8.9 (mean)	1 (month)	onabotulinumtoxinA	Below 100	Subcutaneous	NR	Pain
Yoo et al., 2022 [38]	1	NR	Intervention: BoNT-A (Nabota) with levobupivacaine; Control: levobupivacaine	75	LSB	Fluoroscopy	Temperature, pain, velocity of popliteal artery, cold intolerance questionnaire, patient’s global impression of change, adverse events

BoNT-A: botulinum neurotoxin type A; BoNT-B: botulinum neurotoxin type B; CMAP: compound muscle action potential; LSB: lumbar sympathetic block; NR: not reported.

**Table 3:** Summary of data extracted from included studies.

Quality Assessment						Summary of Findings, WMD/OR (95% CI)		
Number of Participants (Studies)	Risk of Bias	Inconsistency	Indirectness	Imprecision	Publication Bias	Control	Botulinum Neurotoxin	Certainty of Evidence
Pain 3 weeks to 1 months: 62 (3)	Serious limitation ^a^	No serious limitation	No serious limitation ^c^	Serious limitation ^d^	Undetectable	- ^e^	WMD −1.036 (−1.673, −0.400)	Low ⨁⨁◯◯ ^f^
Pain 2 to 3 months: 52 (2)	Serious limitation ^a^	Serious limitation ^b^	No serious limitation ^c^	Serious limitation ^d^	- ^e^	- ^e^	WMD −0.895 (−2.249, 0.458)	Very Low ⨁◯◯◯ ^f^
Risk of adverse events: 54 (2)	Serious limitation ^a^	No serious limitation	No serious limitation ^c^	Serious limitation ^d^	- ^e^	- ^e^	OR 0.698 (0.136, 3.581)	Low ⨁⨁◯◯ ^f^

CI: confidence interval; WMD: weighted mean difference. ^a^ Only one randomized controlled trial included scored all low risk of bias. ^b^ The I^2^ was >50%. ^c^ No indirectness was noted. ^d^ The upper and lower limit of 95% CI pointed in opposite directions or fell on different sides of the clinical significant cutoff point. ^e^ There were insufficient data available so far. ^f^ ⨁◯◯◯ means very low, ⨁⨁◯◯ means low.

## Data Availability

No new data were created in this study.

## Supplementary Materials

The following supporting information can be downloaded at: https://www.mdpi.com/article/10.3390/life12122037/s1, File S1: PRISMA checklist and search strategies for meta-analysis.
